# Using #ActuallyAutistic on Twitter for Precision Diagnosis of Autism Spectrum Disorder: Machine Learning Study

**DOI:** 10.2196/52660

**Published:** 2024-02-14

**Authors:** Aditi Jaiswal, Peter Washington

**Affiliations:** 1 Department of Information and Computer Sciences University of Hawaii at Manoa Honolulu, HI United States

**Keywords:** autism, autism spectrum disorder, machine learning, natural language processing, public health, sentiment analysis, social media analysis, Twitter

## Abstract

**Background:**

The increasing use of social media platforms has given rise to an unprecedented surge in user-generated content, with millions of individuals publicly sharing their thoughts, experiences, and health-related information. Social media can serve as a useful means to study and understand public health. Twitter (subsequently rebranded as “X”) is one such social media platform that has proven to be a valuable source of rich information for both the general public and health officials. We conducted the first study applying Twitter data mining to autism screening.

**Objective:**

We aimed to study the feasibility of autism screening from Twitter data and discuss the ethical implications of such models.

**Methods:**

We developed a machine learning model to attempt to distinguish individuals with autism from their neurotypical peers based on the textual patterns from their public communications on Twitter. We collected 6,515,470 tweets from users’ self-identification with autism using “#ActuallyAutistic” and a separate control group. To construct the data set, we targeted English-language tweets using the search query “#ActuallyAutistic” posted from January 1, 2014 to December 31, 2022. We encrypted all user IDs and stripped the tweets of identifiable information such as the associated email address prior to analysis. From these tweets, we identified unique users who used keywords such as “autism” OR “autistic” OR “neurodiverse” in their profile description and collected all the tweets from their timelines. To build the control group data set, we formulated a search query excluding the hashtag “#ActuallyAutistic” and collected 1000 tweets per day during the same time period. We trained a word2vec model and an attention-based, bidirectional long short-term memory model to validate the performance of per-tweet and per-profile classification models. We deleted the data set and the models after our analysis.

**Results:**

Our tweet classifier reached a 73% accuracy, a 0.728 area under the receiver operating characteristic curve score, and an 0.71 *F*_1_-score using word2vec representations fed into a logistic regression model, while the user profile classifier achieved an 0.78 area under the receiver operating characteristic curve score and an *F*_1_-score of 0.805 using an attention-based, bidirectional long short-term memory model.

**Conclusions:**

We have shown that it is feasible to train machine learning models using social media data to predict use of the #ActuallyAutistic hashtag, an imperfect proxy for self-reported autism. While analyzing textual differences in naturalistic text has the potential to help clinicians screen for autism, there remain ethical questions that must be addressed for such research to move forward and to translate into the real world. While machine learning has the potential to improve behavioral research, there are still a plethora of ethical issues in digital phenotyping studies using social media with respect to user consent of marginalized populations. Achieving this requires a more inclusive approach during the model development process that involves the autistic community directly in the ideation and consent processes.

## Introduction

Millions of individuals are autistic. A core complexity of autism lies in its dynamic profile that changes with age, often leading to the misattribution of behavioral characteristics to other conditions such as anxiety and obsessive-compulsive disorder [1,2]. Unfortunately, there are limitations on the availability of standard tests [3], leading to misdiagnoses or delayed services [4], often leading to negative outcomes later in life [5]. Social media has been proposed as a means for real-time public health monitoring, offering insights into individuals’ thoughts, emotions, behaviors, and daily struggles. Such nonclinical data can potentially enable clinicians and researchers to develop early screening tools in a less invasive manner. This digital footprint can be analyzed to study the linguistic characteristics of autism and other developmental delays [6]. However, this potential for social good may be outweighed by the salient possibility of harm.

In recent years, social media has emerged as a promising tool for mining behavioral and observational data. The collection of digital data from social media, wearable devices, and smartphones holds potential for improving health care. Research in mental health, such as identifying depression and mood changes [7-13] and real-time mapping of natural disasters [14,15] or infectious disease spread and its effect on emotional health [16-23] has greatly benefited from such “digital phenotyping” studies. Among social media platforms, Twitter (subsequently rebranded as “X”), known for its concise microblogging nature with tweets limited to 280 characters, has emerged as a valuable source of personalized data, boasting an active monthly user base of around 450 million individuals [24].

Autism has been the subject of multiple clinical trials, reviews, and epidemiological studies conducted using behavioral features such as eye gaze [25], prosody [26], asynchronous body movement [27], facial expressions [28,29], mobile phone data [30-33], or even electroencephalograms [34]. However, only a handful of studies have used social analytical tools [35-38], especially Twitter [39-41], for investigating autism. In addition, other social networking sites such as Reddit [42-45], Facebook [46], Instagram [47,48], Flickr [49], and Sina Weibo [50] have also provided a valuable source of data for detecting and studying mental health conditions, substance abuse, and risky behaviors. Using these previous works as inspiration, we curated a novel, extensive Twitter data set to study various aspects of social communication that differentiate people with autism from their neurotypical peers on a larger scale than previous work.

Our goal was to build a classifier to aid in affordable autism screening using Twitter data, enabling support for communities with limited access to diagnostic resources. While we were able to build such a model with reasonable predictive power for a first pass at this task, we note that we did not obtain explicit consent from the study population. We therefore deleted all the data and models that we developed after the completion of our analysis. Given the potential of such research to harm user privacy and the lack of consent, we discuss the ethical implications of this research. We note that the availability of the resulting models has the potential to promote unethical practices that can occur for more malicious purposes, such as profiling of individuals by medical insurance companies, use by colleges to assess applicants, and surveillance of citizens by governments. We therefore caution researchers and practitioners against building such models without obtaining explicit consent and practicing participatory community-centered research prior to model development.

## Methods

### Overview

**Figure 1:**
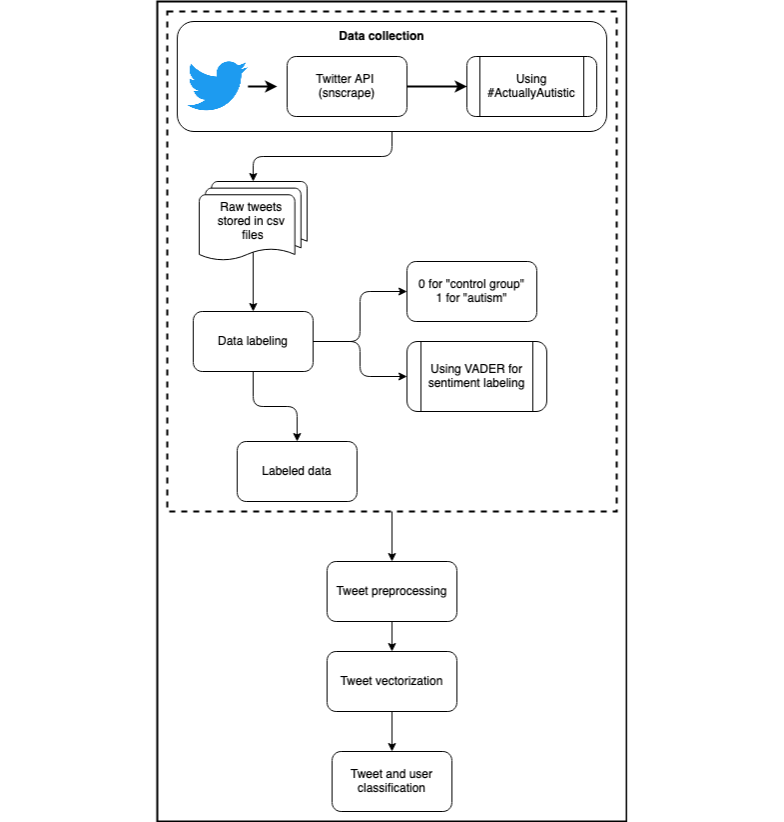
Pipeline for the creation of the novel Twitter (subsequently rebranded as “X”) autism data set. API: application programming interface; VADER: Valence Aware Dictionary for Sentiment Reasoning.

Here, we describe the data set curation process (Figure 1), preprocessing steps, and a series of analyses on the curated data. We started by analyzing the sentiments and topics within the data set to discover some qualitative insights. We then performed per-tweet and per-user classifications of autism to understand the linguistic differences between the users in the autism and control groups.

### Data Collection

In recent years, hashtags such as #MeToo, #BlackLivesMatter, and #StopAsianHate have played significant roles in promoting social movements and campaigns, including those aimed at raising awareness about specific societal issues. Within the autism community, popular hashtags such as #AutismMom and #AutismParent have represented the perspectives of neurotypical parents, significantly influencing research and policies in this domain. However, these advocacy groups often overshadow adults with autism, creating a gap in their representation within decision-making processes. To address this issue, a paradigm shift occurred in the autism rights movement through the hashtag “#ActuallyAutistic” [51,52]. This movement has emphasized understanding the experiences, challenges, and perspectives of individuals on the autism spectrum, redirecting attention toward them rather than solely focusing on caregivers.

Using the hashtag as the criteria for our corpus selection, we extracted Twitter conversations of users self-identifying with autism to study the differences in their linguistic patterns. Our data extraction involved using *snscrape* (JustAnotherArchivist) [53], a Python-based library allowing social media scraping without requiring personal Twitter application programming interface keys and providing powerful search functionality to help filter tweets based on various conditions, such as date-time, language, or location. We targeted English-language tweets using “#ActuallyAutistic” posted from January 1, 2014, to December 31, 2022. To identify users self-identifying with autism, we searched for keywords such as “autism,” “autistic,” or “neurodiverse” within their profile descriptions (bios). Additionally, we considered usernames and tweet contents for users who used these keywords solely in their usernames. Finally, we extracted all the tweets from the timelines of these users to construct the autism data set, which consists of 3,137,952 tweets from 17,323 individuals. Associated metadata such as username, account created, friend count, and date of tweets posted were also extracted and could be used for statistical or network analysis.

To build a tweet classifier for individuals with autism and their neurotypical peers, we collected a sample of random tweets as part of the control group. To achieve this, we formulated a search query excluding the hashtag, “-#ActuallyAutistic,” using the advanced query searching operators and methods provided by Dr Igor Brigadir [54]. However, this approach carries the risk of data leakage, whereby users who have not posted any autism-related content may possess autism-related keywords in their profile description or username. To avoid this, we screened users who had any such keywords in their profile description or usernames, or who were also present in the autism data set, and subsequently removed them from the sample. We collected 1000 control tweets per day during the same time period to obtain a total of 3,377,518 tweets across 171,273 individual users.

### Data Labeling

To train a supervised machine learning model effectively, labeled data that associate each data point with a respective class are crucial. We automatically labeled the tweets from the autism data set as belonging to the class “autism,” assigned label 1. All other tweets from the control group data set were labeled as belonging to the class “control group,” assigned label 0. However, it is important to clarify that these tweet labels were used temporarily for classification purposes and were not permanently stored in the data set. It is important to note that obtaining ground-truth labels can be a costly and time-consuming process, and the performance of machine learning models is often found to decrease with a decrease in labeled data set size. Weak supervision approaches leverage partially accurate or noisy sources for annotations, which can be more efficient than manual labeling.

### Data Preprocessing

Working with raw, unstructured Twitter data is challenging because the conversational text contains too many noisy elements, such as punctuation, abbreviations, emojis, and other stray characters. Thus, before using such data for model training, it is necessary to clean and preprocess the data, which is an essential step for any natural language processing task. We started by removing the usage of any profane language in the tweets, such as cursing or swear words, using a Python library called *better-profanity* [55], which is designed to flag inappropriate words using string comparison and mask them using special characters (the default setting uses “*”). While profane language can sometimes be highly emotive and help in understanding the sentiments of a text, we chose to censor any such words while classifying the tweets, as such words can be used by any individual and might not help in classification tasks. However, we considered the contribution of profane language through sentiment analysis and observed that the polarity of the sentiments was almost similar when using clean and uncensored tweets.

We then tokenized the text into words; removed any nonalphanumeric characters, hyperlinks, user mentions, and HTML tags; and converted the word tokens into lower case to avoid any confusion and data redundancy. We removed stop words to avoid adding noise and complexity to the features with no meaningful information. To further simplify the input space and normalize the vocabulary, we applied stemming and lemmatization. We also removed any hashtags or a list of keywords related to autism such as “actuallyautistic,” “autism,” “autistic,” “autismacceptance,” “autismawareness,” “askingautistics,” “askingautistic,” “neurodiversity,” “neurodivergent,” “allautistics,” “adhd,” “mentalhealth,” “autism,” “diagnosis,” “autistics,” “autismpride,” and “autismspeaks,” which could introduce bias and lead to model overfitting.

### Sentiment Labeling

We compared the sentiments of tweets posted by individuals with autism against those from the control group in order to understand the subjective characteristics and emotional polarity around the topic. Initially, we conducted sentiment analysis on the original data set, which contained profanity. Additionally, we wanted to explore how profane language can affect the sentiments of the tweets, and thus we also conducted sentiment analysis on a pseudoclean data set after removing any profane words. Sentiment analysis commonly involves 2 approaches: machine learning and lexical. We used the Valence Aware Dictionary for Sentiment Reasoning (VADER) [56], a lexical approach specifically attuned to sentiments expressed in social media or microblogs like context, to analyze the sentiments of the curated data set. VADER has been explicitly trained on social media data sets (such as social media posts or New York Times editorials) and requires no training data. VADER applies a set of rules and heuristics to the sentiment scores of the individual words to determine the overall sentiment of the sentence and returns a dictionary of negative, neutral, positive, and overall (normalized) sentiment scores for the sentence.

### Topic Modeling

The objective of our topic modeling analysis was to investigate whether there exist specific themes and semantic patterns that are frequently discussed in relation to autism and can offer insights beneficial for clinicians and policymakers. Topic modeling is an unsupervised learning technique used to uncover concealed topics and coherent themes within textual data. We used the Top2Vec [57] algorithm, which offers a dynamic approach to discovering topics within a corpus of text data by making use of the spatial proximity of the words.

### Tweet-Level Classification

Our initial focus involved training a model specifically designed to predict autism based on the content within individual tweets. To build this tweet classifier, we identified unique users from both the autism and control data sets, allocating an 85:15 split for training and testing purposes. Data splitting by user rather than by tweet avoids data leakage, where a user’s tweets might scatter across both training and testing sets, potentially leading to overfitting by the model due to learning user-specific patterns. The tweets, with no profanity, were preprocessed as defined in the previous section and formed the training and test sets. The categorical labels, representing whether a tweet belonged to a user in the autism or control group, were used as the basis for model training and evaluation. Additionally, the training data set underwent an 85:15 split, separating it into training and validation subsets, which was used to fine-tune the model and adjust hyperparameters.

For text-to-numeric vectorization, we used 2 approaches: a bag-of-words term frequency–inverse document frequency (TF-IDF) method and word2vec embeddings. We started by training TF-IDF feature representation using various classical machine learning algorithms: support vector machines, naive Bayes, logistic regression, and XGBoost (extreme gradient boosting), using 5-fold cross-validation and accuracy as the primary evaluation metric to identify the best classification method. We then trained the word2vec model using the best-identified algorithm for better feature representation. This approach captures both semantic and syntactic similarities among words, and we assessed its efficacy using a more comprehensive array of evaluation metrics.

### User Profile Classification

Our subsequent task involved training a model to predict autism by considering all tweets from an individual user’s timeline. To ensure a more representative data set and prevent potential model overfitting, we isolated unique users who had shared a minimum of 5 tweets and split them into an 80:20 ratio for training and testing. The preprocessed tweets from each user were then grouped together to form an individual document. For model training, we used an attention-based, bidirectional long short-term memory (Bi-LSTM) model vectorized with a randomly initialized, self-trained embedding layer. As the tweets vary in their lengths and raw text cannot be directly represented as dense vectors in the way that images can, we used padding and an extra “unknown” token during tokenization to achieve the fixed length input and represent any unseen tokens.

### Ethical Considerations

While social media data can help with public health analysis by offering a less intrusive and real-time monitoring approach for disease symptomatology and public sentiments, it also poses ethical challenges by exposing the users to harm or the potential leaking of personally identifiable information. First, this study was approved by all ethics-related regulatory bodies at the University of Hawaii. The study has been approved by the University of Hawaii Institutional Review Board (2023-00248) under an expedited review procedure, and the user information was deidentified. We also ran the request through University of Hawaii institutional data governance to approve this study, where it was determined that the study is exempt from further data governance review due to the inherently public nature of the study data. We also took additional measures not required by the Institutional Review Board. Specifically, we encrypted user IDs, reducing the chances of user reidentification. We also anonymized any user mentions or personal information, such as email addresses, contained within the tweets. These steps were aligned with the ethical considerations outlined in various research studies on social media analysis [58-60].

The public nature of social media data can often overshadow participants’ consent, leaving them unaware or unsure of the inclusion of their data in the research. Williams et al [61] observed that 84% of respondents were not at all or only slightly concerned about the use of the Twitter posts for university research. However, this leaves a considerable portion of the population who remains concerned. The conditions and privacy policies for data use are often long, with complex legal terms that the users may fail to understand or authorize, leaving them unaware of the consequences. While it can be impractical to obtain explicit consent on a per-study basis for large-scale social media analytics research, we recommend that the research community find ways to support large-scale consent procedures. This study highlights the need of a regulatory framework for social media data mining.

There remain concerns surrounding the ethics of social media analytics research on individuals with autism. While this study and previous studies typically safeguard user data by deidentifying and anonymizing metadata, there remains a risk of identifying users based on their posted content. This underscores the immediate need for the creation of ethical tools and methodologies that facilitate scientific research based on social media data while adhering to ethical principles. Due to these inherent risks, the data set and the model that we built using those data have been deleted. When the data set did exist, it was never shared outside of the original authors.

## Results

### Data Records

The autism subset, collected from 17,323 self-reported individuals with autism, contains 3,137,952 tweets. The control subset, collected from 171,273 users, consists of 3,377,518 tweets. The combined data set contains the following columns: user ID (a unique value assigned to each Twitter account), profile description (a short summary of the account posted by the user), account created (date-time when the account was created), friends count (number of accounts the user follows), followers count (number of accounts the user is being followed by), tweet date (date-time when the tweet was posted), tweet ID (a unique ID assigned to each tweet), tweet text (original tweet), a list of hashtags present in each tweet, number of replies (number of times the tweet has been replied to), number of retweets (number of times the tweet was retweeted), number of likes the tweet got, and source from where the tweet was posted (web, mobile device, or app). While we focused on using tweet text as the primary source of data, other supporting metadata could be used in the future for network analysis or statistical studies.

### Exploratory Data Analysis

The data set’s columns for hashtags and locations were found to contain the highest number of missing values during our analysis. While not all tweets are accompanied by hashtags or location details, users possess the liberty to input any desired location on their profiles. Our analysis revealed that a large portion of users either did not provide their actual location or had inconsistencies in their location entries. Among the top 20 location values identified, most were variations of “United Kingdom,” such as “UK,” “London, England,” “England, United Kingdom,” and “South East, England.” However, other entries were less informative and included phrases such as “Picnic party” and “My parent’s basement.” Due to the majority of the missing data and to safeguard users’ personal information, we opted to exclude the location column from the data set before using it to train any machine learning algorithms.

Further analysis of the yearly distribution of tweets revealed a rising trend in discussions related to autism across the years. This trend suggests that individuals on the autism spectrum are increasingly embracing social media platforms, potentially opening up numerous employment prospects and serving as an effective channel to educate the public about developmental delays. Additionally, sharing behavioral symptoms through social engagement could be beneficial to others to build better community support. This increased social involvement may hold significance not only in social science [62] but also in human-computer interaction research [63], offering insights to design more inclusive and efficient digital environments.

### Sentiment Analysis

The VADER sentiments of most of the autism and control group tweets were found to be positive and neutral, respectively, as shown in Table 1.

**Figure 2:**
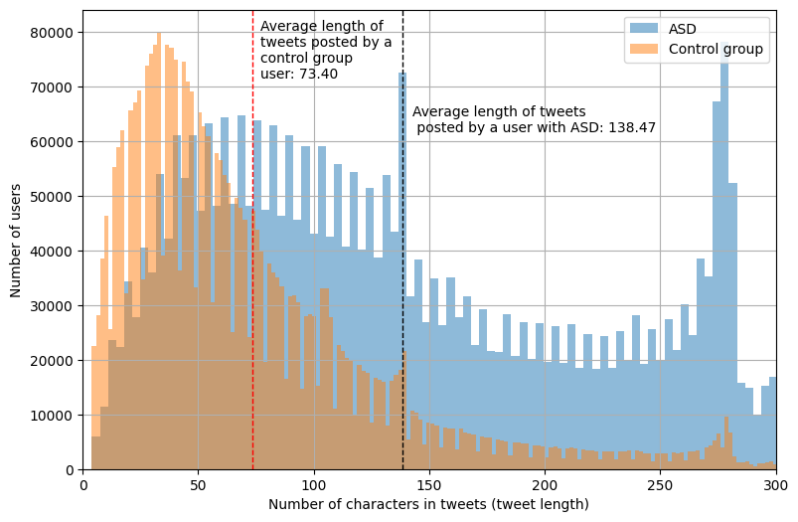
Histograms of number of characters in the tweets for the 2 groups.

This was supported by another interesting observation: tweets from individuals with autism comprised a higher character count compared to those from the control group (Figure 2). The histograms depicting the word counts in tweets from both groups follow similar distributions but with a substantial difference in their means. This disparity strongly suggests varying linguistic patterns between these 2 groups.

**Table 1:** Distribution of sentiments in the autism spectrum disorder and control group data sets.

Data set and VADER^a^ sentiments		Frequency, n (%)
**In original autism tweets (n=3,137,952)**		
	Positive	1,528,183 (48.7)
	Negative	812,730 (25.9)
	Neutral	797,039 (25.4)
**In clean autism tweets (n=3,137,952)**		
	Positive	1,562,700 (49.8)
	Negative	756,247 (24.1)
	Neutral	819,005 (26.1)
**In original control group tweets (n=3,377,518)**		
	Positive	1,280,080 (37.9)
	Negative	938,950 (27.8)
	Neutral	1,158,488 (34.3)
**In clean control group tweets (n=3,377,518)**		
	Positive	1,323,987 (39.2)
	Negative	719,411 (21.3)
	Neutral	1,334,120 (39.5)

^a^VADER: Valence Aware Dictionary for Sentiment Reasoning.

### Topic Modeling

Using just the autism data set, multiple topics were discovered, and the word clouds of a few topics are shown in Multimedia Appendix 1.

As it can be seen, the majority of topics were related to behavioral and emotional symptoms such as “hyperactivity,” “fidgeting,” “depressed,” “anxiety,” “trembling,” and “overwhelmed.” Interestingly, a considerable number of documents also focused on terms such as “vaccine,” “therapy,” “misdiagnosis,” and “cats.” These findings may be attributed to the frequent misdiagnosis or delayed diagnosis of autism, prompting individuals to seek therapy, support, and guidance. The presence of vaccine-related discussions likely stems from misinformation and its negative impact on individuals affected by autism. However, given the time frame in which the data set was collected, it is also possible that these tweets are related to COVID-19 vaccines. Last, multiple studies [64,65] have found that children with autism are more at ease with cats due to their nonintrusive nature, lack of prolonged eye contact, and their ability to alleviate stress and interpret emotional cues.

Deriving specific topics from the control group’s Twitter conversations was challenging given their scattered and diverse nature. Most of these discussions centered around internet personalities, random conversations, specific days of the week, or special occasions such as birthdays and anniversaries. Interestingly, some broader topics related to animals surfaced in these conversations, but not as specifically focused as observed in autistic user conversations—specifically mentioning cats. Some of these posts also displayed the use of emotional words, suggesting that pets or animals may provide therapeutic benefits.

### Technical Validation

The performance metrics for tweet classification are shown in Tables 2 and 3. Table 2 displays the results from TF-IDF feature representations across several classical machine learning models, while Table 3 displays the results using word2vec feature vectors trained with logistic regression. While the TF-IDF vectorization yielded similar accuracy using different machine learning algorithms for tweet classification, logistic regression was chosen as the best predictor due to its superior performance and shorter training time. The results of the word2vec model were found to be consistent with the semantic similarities of the words. For instance, “autism” exhibited higher cosine similarity to terms such as “Aspergers,” “neuroatypical,” and “autism spectrum condition,” indicating the model’s proficiency in capturing semantic relationships between words.

Table 4 displays the results for user classification. Although there is a class imbalance in the number of users with autism versus controls, the attention-based LSTM model still seems to yield better measures, with *F*_1_-scores of 0.7 and 0.9 on the “autism” and “control group” classes, respectively, and an AUC score of 0.78.

**Table 2:** Summary of results obtained for tweet classification from term frequency–inverse document frequency vectorization to identify the best algorithm based on accuracy.

Model	Validation set accuracy
Support vector machine	0.615
Naive Bayes	0.598
Logistic regression	0.63
XGBoost^a^	0.624

^a^XGBoost: extreme gradient boosting.

**Table 3:** Summary of results obtained for tweet classification from the word2vec model using the highest performing model, logistic regression.

Metric performance on test set	Value
Accuracy	0.73
*F*_1_-score	0.71
AUC^a^ score	0.728

^a^AUC: area under the receiver operating characteristic curve.

**Table 4:** Summary of results obtained for user classification from Keras embedding using the attention+Bi-LSTM^a^ model.

Metric performance on test set	Value
*F*_1_-score	0.805
AUC^b^ score	0.78

^a^Bi-LSTM: bidirectional long short-term memory.

^b^AUC: area under the receiver operating characteristic curve.

## Discussion

### Overview

The shift in society’s reliance on social media for information, in contrast to traditional news sources, along with the immense volume of generated data, has resulted in an increased focus on the use of natural language processing for social media analytics. While research tools using facial expressions [6,66-75] and eye gazing for phenotyping autism [76,77] are promising, there exists a current deficiency in standardizing precise methods for assessing deviations from typical social interactions. The *F*_1_-scores of 0.71 in tweet classification and 0.80 in user classification signify substantial semantic distinctions in messages posted by individuals who did and did not post using the #ActuallyAutistic hashtag. Tweets by individuals using the hashtag demonstrated a higher frequency of emotional language, corroborated by the word2vec model’s stronger semantic associations among such words, reinforcing the model’s predictive capability. This finding, coupled with previous studies using computer vision models [76,78], suggests that digital phenotyping using social media could be used to support effective autism screening strategies and to facilitate early detection. Organizations such as the National Institutes of Health are actively funding research [79,80] using data from social media coupled with novel AI–based tools to improve public health surveillance and precision diagnostics, and these organizations are emphasizing the importance of maintaining ethical practices during the process.

We would also like to highlight that any social media analytics research should always be supported by ethical practices and an adherence to user privacy. As user data on social media platforms are often openly available, it is critical to obtain user consent when building AI models for marginalized communities. Without consent, use of the data may put some marginalized communities at risk of data leakage or potential misuse of their data. In the past, there have also been numerous cases of public data being used for training large language models (LLMs) without informed consent. While LLMs have revolutionized the field of AI, such practices highlight the need for regulations in the consent process and updating users about the use of their data in a simple and transparent manner. This study has helped us learn more about the use of social media for different AI-based research and the urgent need to integrate the community in the research process. Such integration will not only lead to an effective early screening tool but will also enable the maintenance of an ethical, privacy-protected system.

### Limitations

There are certain limitations to consider in this study. While we focused on individuals who self-identified as autistic, there is no clinical validation for their diagnosis. Annotations from clinical experts or crowdsourcing can help. Furthermore, there is a possibility of data leakage, where the identified users may not be autistic but instead could be family members, parents, caregivers, or advocacy organizations belonging to a different study population and still using the hashtags. However, the frequency of this type of leakage is predicted to be rare due to the negative social connotations of using #ActuallyAutistic without a diagnosis. There might also be a possibility of some data leakage of an individual with autism falling into the control group, but with autism having a prevalence rate of <3%, the model performance should not degrade by more than 3% if an individual who chose not to self-identify themselves with autism falls into the control group cohort. The predictive power of social media is not to be used at an individual level but at a broader cross-sectional level, possibly combined with self-reported questionnaires for enhanced accuracy in neurological studies.

In addition, the sentiment polarity obtained through VADER may lack accuracy compared to human-labeled sentiments, as human sentiments are influenced by various factors such as surroundings and politics, making reliable labeling challenging. Moreover, this study only considered the English language, potentially missing out on information from other countries or languages that could aid the model in making better predictions. This also raises concerns about the lack of diversity in the data [81], where only English-speaking users from higher socioeconomic groups or younger adults are represented in the data set, as they comprise a larger portion of Twitter users.

Nevertheless, the primary limitation of this study is that we were unable to obtain explicit consent from our study population. Because of this limitation, we have deleted the models and the data set, and we highlight the potential misuses of this model by more malicious actors. For example, the model could be used for admissions decisions to universities, hiring decisions, government surveillance programs, or even more nefarious purposes. We keep this paper as a case study of what is currently possible with publicly available social media data, and we encourage the research community and other AI innovators to think throughly about protections against harm against marginalized communities.

### Future Work

We recommend that the research community pause before conducting further research on social media–based predictive models for autism. Interesting avenues for future work include (1) developing strategies for obtaining explicit consent on a large scale on social media and (2) conducting surveys of the autistic community to understand whether and how social media analytics may be useful.

## Appendix

Multimedia Appendix 1 Topics observed in autism spectrum disorder data set using the Top2Vec algorithm.

## Abbreviations

AUC: area under the receiver operating characteristic curve
Bi-LSTM: bidirectional long short-term memory
TF-IDF: term frequency–inverse document frequency
VADER: Valence Aware Dictionary for Sentiment Reasoning
XGBoost: extreme gradient boosting
