# Addendum: Using #ActuallyAutistic on Twitter for Precision Diagnosis of Autism Spectrum Disorder: Machine Learning Study

**DOI:** 10.2196/59349

**Published:** 2024-07-17

**Authors:** Aditi Jaiswal, Aekta Shah, Christopher Harjadi, Erik Windgassen, Peter Washington

**Affiliations:** 1 Department of Information and Computer Sciences University of Hawaii at Manoa Honolulu, HI United States; 2 Salesforce San Francisco, CA United States; 3 Department of Computer Science University of California, Berkeley Berkeley, CA United States; 4 Department of Computer Science and Engineering University of California, Riverside Riverside, CA United States

The authors of “Using #ActuallyAutistic on Twitter for Precision Diagnosis of Autism Spectrum Disorder: Machine Learning Study” (JMIR Form Res 2024;8:e52660) have updated the narrative throughout the paper to highlight ethical concerns with social media scraping without user consent, particularly in cases of data belonging to vulnerable populations such as the autism community. The authors have also updated the text to emphasize that both the data collected and the artificial intelligence (AI) models that were trained were deleted and never shared with anyone.

The *Objective, Methods, Results,* and *Conclusions* subsections of the *Abstract* have been revised as follows:

**Objective**

We aimed to study the feasibility of autism screening from Twitter data and discuss the ethical implications of such models.

**Methods**

We developed a machine learning model to attempt to distinguish individuals with autism from their neurotypical peers based on the textual patterns from their public communications on Twitter. We collected 6,515,470 tweets from users’ self-identification with autism using “#ActuallyAutistic” and a separate control group. To construct the data set, we targeted English-language tweets using the search query “#ActuallyAutistic” posted from January 1, 2014 to December 31, 2022. We encrypted all user IDs and stripped the tweets of identifiable information such as the associated email address prior to analysis. From these tweets, we identified unique users who used keywords such as “autism” OR “autistic” OR “neurodiverse” in their profile description and collected all the tweets from their timelines. To build the control group data set, we formulated a search query excluding the hashtag “#ActuallyAutistic” and collected 1000 tweets per day during the same time period. We trained a word2vec model and an attention-based, bidirectional long short-term memory model to validate the performance of per-tweet and per-profile classification models. We deleted the data set and the models after our analysis.

**Results**

Our tweet classifier reached a 73% accuracy, a 0.728 area under the receiver operating characteristic curve score, and an 0.71 *F*_1_-score using word2vec representations fed into a logistic regression model, while the user profile classifier achieved an 0.78 area under the receiver operating characteristic curve score and an *F*_1_-score of 0.805 using an attention-based, bidirectional long short-term memory model.

**Conclusions**

We have shown that it is feasible to train machine learning models using social media data to predict use of the #ActuallyAutistic hashtag, an imperfect proxy for self-reported autism. While analyzing textual differences in naturalistic text has the potential to help clinicians screen for autism, there remain ethical questions that must be addressed for such research to move forward and to translate into the real world. While machine learning has the potential to improve behavioral research, there are still a plethora of ethical issues in digital phenotyping studies using social media with respect to user consent of marginalized populations. Achieving this requires a more inclusive approach during the model development process that involves the autistic community directly in the ideation and consent processes.

The first paragraph of the *Introduction* has been revised as follows:

Millions of individuals are autistic. A core complexity of autism lies in its dynamic profile that changes with age, often leading to the misattribution of behavioral characteristics to other conditions such as anxiety and obsessive-compulsive disorder [1,2]. Unfortunately, there are limitations on the availability of standard tests [3], leading to misdiagnoses or delayed services [4], often leading to negative outcomes later in life [5]. Social media has been proposed as a means for real-time public health monitoring, offering insights into individuals’ thoughts, emotions, behaviors, and daily struggles. Such nonclinical data can potentially enable clinicians and researchers to develop early screening tools in a less invasive manner. This digital footprint can be analyzed to study the linguistic characteristics of autism and other developmental delays [6]. However, this potential for social good may be outweighed by the salient possibility of harm.

The fourth paragraph of the *Introduction* has been revised as follows:

Our goal was to build a classifier to aid in affordable autism screening using Twitter data, enabling support for communities with limited access to diagnostic resources. While we were able to build such a model with reasonable predictive power for a first pass at this task, we note that we did not obtain explicit consent from the study population. We therefore deleted all the data and models that we developed after the completion of our analysis. Given the potential of such research to harm user privacy and the lack of consent, we discuss the ethical implications of this research. We note that the availability of the resulting models has the potential to promote unethical practices that can occur for more malicious purposes, such as profiling of individuals by medical insurance companies, use by colleges to assess applicants, and surveillance of citizens by governments. We therefore caution researchers and practitioners against building such models without obtaining explicit consent and practicing participatory community-centered research prior to model development.

The second and third paragraphs of the *Ethical Considerations* subsection of the *Methods* section have been replaced with the following paragraphs:

The public nature of social media data can often overshadow participants’ consent, leaving them unaware or unsure of the inclusion of their data in the research. Williams et al [61] observed that 84% of respondents were not at all or only slightly concerned about the use of the Twitter posts for university research. However, this leaves a considerable portion of the population who remains concerned. The conditions and privacy policies for data use are often long, with complex legal terms that the users may fail to understand or authorize, leaving them unaware of the consequences. While it can be impractical to obtain explicit consent on a per-study basis for large-scale social media analytics research, we recommend that the research community find ways to support large-scale consent procedures. This study highlights the need of a regulatory framework for social media data mining.

There remain concerns surrounding the ethics of social media analytics research on individuals with autism. While this study and previous studies typically safeguard user data by deidentifying and anonymizing metadata, there remains a risk of identifying users based on their posted content. This underscores the immediate need for the creation of ethical tools and methodologies that facilitate scientific research based on social media data while adhering to ethical principles. Due to these inherent risks, the data set and the model that we built using those data have been deleted. When the data set did exist, it was never shared outside of the original authors.

The first paragraph of the *Discussion* section has been divided into 2 separate paragraphs as follows:

The shift in society’s reliance on social media for information, in contrast to traditional news sources, along with the immense volume of generated data, has resulted in an increased focus on the use of natural language processing for social media analytics. While research tools using facial expressions [6,66-75] and eye gazing for phenotyping autism [76,77] are promising, there exists a current deficiency in standardizing precise methods for assessing deviations from typical social interactions. The F 1-scores of 0.71 in tweet classification and 0.80 in user classification signify substantial semantic distinctions in messages posted by individuals who did and did not post using the #ActuallyAutistic hashtag. Tweets by individuals using the hashtag demonstrated a higher frequency of emotional language, corroborated by the word2vec model’s stronger semantic associations among such words, reinforcing the model’s predictive capability. This finding, coupled with previous studies using computer vision models [76,78], suggests that digital phenotyping using social media could be used to support effective autism screening strategies and to facilitate early detection. Organizations such as the National Institutes of Health are actively funding research [79,80] using data from social media coupled with novel AI–based tools to improve public health surveillance and precision diagnostics, and these organizations are emphasizing the importance of maintaining ethical practices during the process.

We would also like to highlight that any social media analytics research should always be supported by ethical practices and an adherence to user privacy. As user data on social media platforms are often openly available, it is critical to obtain user consent when building AI models for marginalized communities. Without consent, use of the data may put some marginalized communities at risk of data leakage or potential misuse of their data. In the past, there have also been numerous cases of public data being used for training large language models (LLMs) without informed consent. While LLMs have revolutionized the field of AI, such practices highlight the need for regulations in the consent process and updating users about the use of their data in a simple and transparent manner. This study has helped us learn more about the use of social media for different AI-based research and the urgent need to integrate the community in the research process. Such integration will not only lead to an effective early screening tool but will also enable the maintenance of an ethical, privacy-protected system.

The following paragraph has been added as the final paragraph of the *Limitations* subsection of the *Discussion* section:

Nevertheless, the primary limitation of this study is that we were unable to obtain explicit consent from our study population. Because of this limitation, we have deleted the models and the data set, and we highlight the potential misuses of this model by more malicious actors. For example, the model could be used for admissions decisions to universities, hiring decisions, government surveillance programs, or even more nefarious purposes. We keep this paper as a case study of what is currently possible with publicly available social media data, and we encourage the research community and other AI innovators to think throughly about protections against harm against marginalized communities.

The *Future Work* subsection of the *Discussion* has been rewritten as follows:

We recommend that the research community pause before conducting further research on social media–based predictive models for autism. Interesting avenues for future work include (1) developing strategies for obtaining explicit consent on a large scale on social media and (2) conducting surveys of the autistic community to understand whether and how social media analytics may be useful.

A citation to reference 82 (Maenner MJ, Shaw KA, Bakian AV, Bilder DA, Durkin MS, Esler A, et al. Prevalence and characteristics of autism spectrum disorder among children aged 8 years - autism and developmental disabilities monitoring network, 11 sites, United States, 2018. MMWR Surveill Summ. 2021;70(11):1-16) was also removed from the *Future Work* section. Consequently, the total number of references changed from 83 to 82, and reference 83 was renumbered as 82.

Finally, the authors have revised the term “ASD” to “autism” throughout the paper.

The correction will appear in the online version of the paper on the JMIR Publications website on July 17, 2024, together with the publication of this correction notice. Because this addendum was made after submission to PubMed, PubMed Central, and other full-text repositories, the corrected article has also been resubmitted to those repositories.

## Data Availability

The data set curated for this research as well as the models that were built using this data set have been deleted. We want to emphasize that neither the data set nor the models were ever shared with anyone outside of the original authors of the paper, and the authors no longer have access to these deleted data and models.

