# Supplementary figures and images for: Using #ActuallyAutistic on Twitter for Precision Diagnosis of Autism Spectrum Disorder: Machine Learning Study
Source: JMIR Form Res. 2024 Feb 14;8:e52660. doi: 10.2196/52660 (PMC10902768; doi:10.2196/52660)

**Figure S1**. Topics observed in autism spectrum disorder data set using Top2Vec algorithm.


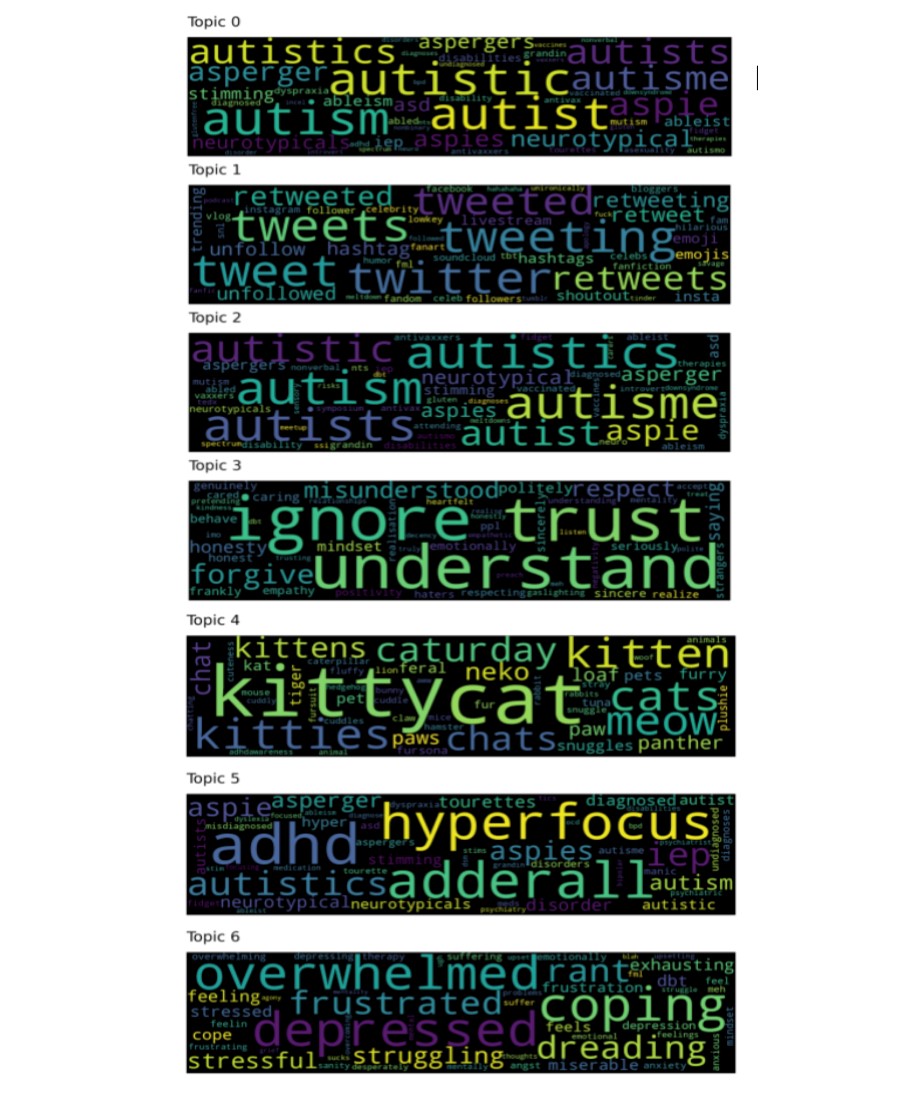

Supplement: Multimedia Appendix 1 [file formative_v8i1e52660_app1.docx]
